# Gastrointestinal neoplasia: carcinogenic interaction between bile acids and *Helicobacter* *pylori* in the stomach

**DOI:** 10.1172/JCI160194

**Published:** 2022-05-16

**Authors:** Madeline Alizadeh, Jean-Pierre Raufman

**Affiliations:** 1 Institute for Genome Sciences and; 2 Department of Medicine, Division of Gastroenterology and Hepatology, University of Maryland School of Medicine, Baltimore, Maryland, USA.; 3 VA Maryland Healthcare System, Baltimore, Maryland, USA.; 4 Marlene and Stewart Greenebaum Comprehensive Cancer Center and; 5 Department of Biochemistry and Molecular Biology, University of Maryland School of Medicine, Baltimore, Maryland, USA.

## Abstract

Bile acids modulate cell functions in health and disease, however, the mechanisms underlying their actions on neoplastic cells in the gastrointestinal (GI) tract remain largely unknown. In this issue of the *JCI*, Noto et al. comprehensively analyzed how interactions between *Helicobacter pylori* infection, iron deficiency, and bile acids modulate gastric inflammation and carcinogenesis. The investigators used sophisticated models, including INS-GAS mice with elevated serum gastrin and gastric acid secretion, in which *H*. *pylori* infection mimics human disease progression, to show that selected bile acids potentiated the carcinogenic effects of *H*. *pylori* infection and iron depletion. This elegant work has broad translational implications for microbe-associated GI neoplasia. Importantly, bile acid sequestration robustly attenuated the combined effects of *H*. *pylori* infection and iron depletion on gastric inflammation and cancer.

## Bile acids as signaling molecules

Traditionally, bile acids were considered metabolic byproducts of cholesterol metabolism whose sole function was to emulsify fats, thereby aiding their digestion and absorption. Over the past 25 years, the role of bile acids expanded greatly to include prominent functions as signaling molecules within the gut (1) and other organs (2). We now appreciate that bile acids may interact functionally with disparate classes of nuclear and plasma membrane receptors and ion transporters expressed by normal and neoplastic cells, thereby modulating important cell functions in health and disease, including gastrointestinal (GI) neoplasia (3). These receptors include the nuclear farnesoid X (FXR), pregnane X, and vitamin D receptors and at least four families of plasma membrane GPCRs, including the formyl peptide and muscarinic receptors (4), Takeda G-coupled receptor 5 (TGR5) (5), and sphingosine 1-phosphate receptor 2 (S1PR2) (6). Notably, muscarinic receptors appear to play prominent roles in GI neoplasia (7) and, specifically, gastric adenocarcinoma (8, 9).

## Bile acids and GI cancer

Despite a reduced incidence of several GI cancers following the initiation of effective strategies for *Helicobacter* *pylori* eradication, colon cancer and Barrett’s esophagus screening, and other prophylactic interventions, the prognosis for advanced cancers of the esophagus, stomach, biliary tree, pancreas, and colon remains dismal: five-year survival rates are substantially lower than 20%. As discussed recently, bile acids in the GI lumen are ideally positioned to interact with normal and neoplastic epithelial cells as well as gut microbes, immune cells, and enteric neurons within the tumor microenvironment (10). Besides the association with gastric adenocarcinoma (11–13), bile acids are also implicated in the development and progression of esophageal adenocarcinoma (6, 14), cholangiocarcinoma (15), pancreatic ductal adenocarcinoma (16), and colon adenocarcinoma (17). Despite involvement with many cancer types, relatively little is known regarding the molecular mechanisms that regulate bile acid effects on neoplastic cells in the GI tract.

## *H. pylori* plus iron deficiency augment gastric cancer

*H. pylori* is recognized by the WHO as a class 1 carcinogen (i.e., there is strong evidence that prolonged inflammation caused by infection with this microorganism causes gastric cancer). Nonetheless, the outcomes of *H*. *pylori* infection vary greatly, depending on host and *H*. *pylori* strain genetic and environmental factors. Previous work from the Peek laboratory, published in the *JCI* (18), addressed the question of why relatively few of the many individuals infected with *H*. *pylori* develop gastric adenocarcinoma. They showed that iron depletion in Mongolian gerbils infected with a cytotoxin-associated gene A–positive (*cag*-positive) strain of *H*. *pylori* accelerated gastric inflammation and neoplasia by a mechanism involving enhanced assembly of Cag type IV secretion system pili, translocation of CagA into gastric epithelial cells, and IL-8 production (18). Likewise, *H*. *pylori* strains isolated from humans with low ferritin levels induced more robust proinflammatory responses, providing additional evidence that iron deficiency enhances *H*. *pylori* virulence (18).

In their current, scientifically rigorous work in this issue of the *JCI*, Noto et al. (19) greatly extended their previous work by using INS-GAS mice to confirm that iron deficiency exacerbates *H*. *pylori*–induced gastritis and dysplasia and elucidated the underlying mechanisms. INS-GAS mice have mild hypergastrinemia and, by one to three months of age, manifest increased parietal cell numbers and gastric acid secretion, reflecting the trophic and acid-stimulatory effects of gastrin. Replicating the clinical pattern seen in humans, over the long term, these mice develop age-dependent loss of parietal cells with hypochlorhydria, gastric atrophy, metaplasia, and dysplasia leading to invasive gastric cancer by 20 months of age; notably, *H*. *pylori* infection hastens this outcome to seven months of age. Of course, no animal model can fully mimic human disease, but this accelerated progression from *H*. *pylori* infection to gastric neoplasia was a major experimental benefit of using INS-GAS mice.

## Bile acids plus *H*. *pylori* promote gastric cancer

Using an unbiased metabolomics approach to study gastric tissues isolated from uninfected and *H*. *pylori*–infected C57BL/6 and INS-GAS mice, Noto et al. found that, compared with infected mice under iron-replete conditions, variants of muricholic acid, cholic acid, and deoxycholic acid (DCA) were markedly increased by *H*. *pylori* infection in iron-deficient animals (19). As proof of principle, treating INS-GAS mice with DCA augmented, while bile acid sequestration attenuated, *H*. *pylori*–induced gastric inflammation and injury (19). As a measure of clinical relevance, using a retrospective database analysis, the authors found that the use of bile acid sequestrants in humans was associated with reduced gastric cancer risk, and expression of a bile acid receptor, TGR5, paralleled disease severity. Nonetheless, the levels of *Tgr5* expression in mice did not alter the severity of *H*. *pylori*–induced gastric inflammation, suggesting other mechanisms were at play (19). For example, the authors found that coculturing *H*. *pylori* with DCA increased the translocation of CagA into host cells by a yet obscure mechanism (19). Notably, TGR5 is not the only bile acid receptor whose expression may be relevant to gastric cancer; for example, other investigators reported a functional increase in M_3_ subtype muscarinic receptor (M_3_R) expression in gastric adenocarcinoma (8, 9). Moreover, the conclusions in Noto et al. were drawn from somewhat artificial experimental conditions and will require rigorous functional validation in physiological settings, where putative receptors are exposed to relevant concentrations of native and conjugated bile acids for adequate exposure times. Hence, in the context of *H*. *pylori* infection and iron deficiency, a broader exploration of the role played by bile acid interaction with other receptors expressed in gastric neoplasia appears warranted.

## Bile acids and gut microbes promote gastric cancer

Evidence in animal models supports the concept that increased retrograde bile acid spillage into the stomach, so-called bile reflux, promotes gastric neoplasia (11–13). Likewise, several lines of evidence in murine colon cancer models support the concept that increased antegrade spillage of bile acids into the colon promotes colon neoplasia, effects uncovered using mouse strains deficient in the key transporter for small intestinal uptake of bile acids (the apical sodium–bile acid transporter) and a key brake on hepatic bile acid synthesis (FGF15; refs. 20, 21). To our knowledge, whether such bile acid effects are modulated by changes in the gut microbiome has not been explored. This concept is important, and several lines of evidence suggest that specific gut microbes, or a community thereof, may promote colon carcinogenesis (22).

**Figure 1:**
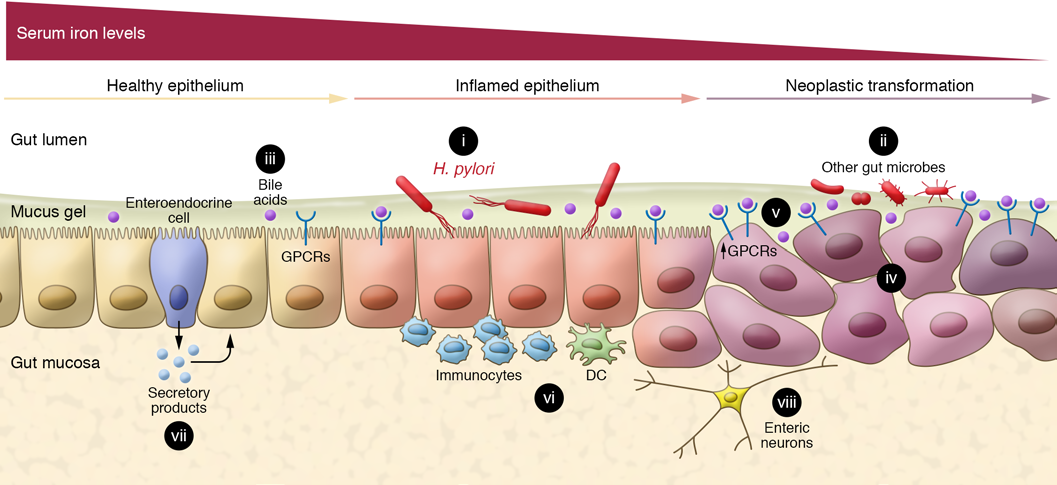
In the setting of auspicious host and bacterial genetic features and a favorable microenvironment, gastric *H*. *pylori* infection promotes chronic inflammation, epithelial cell dysplasia, and neoplastic transformation. (i) In the mucus gel adherent to the surface of gastric epithelial cells, *cag*-positive *H*. *pylori* with the type IV secretory system and additional virulence attributes (e.g., vacuolating cytotoxin) intoxicate the host cell cytosol, thereby activating multiple signaling pathways that control cell proliferation and polarity and other proneoplastic attributes. Notably, *H*. *pylori*–induced gastric mucosal inflammation and the progression to dysplasia and cancer are modulated by dietary micronutrients. As shown by Noto et al. (18, 19), serum iron depletion hastens the development of *H*. *pylori*–induced gastric inflammation and cancer. (ii) Gut microbes in other compartments of the GI tract may promote mucosal neoplasia by mechanisms like those of *H*. *pylori*. (iii) Gastric reflux of bile acids, modified by gut bacterial dehydroxylation and deconjugation, potentiates the proinflammatory and proneoplastic effects of *H*. *pylori* infection. (iv) Gastric cancer cells display intratumor heterogeneity and overexpress receptors for neurotransmitters and bioactive molecules, such as bile acids. (v) Healthy GI epithelial cells express and cancer cells overexpress a variety of GPCRs, including the bile acid receptors TGR5, M_3_R, and S1PR2. (vi) *H*. *pylori* infection is associated with localized immune cell infiltrates in the proliferative zones of gastric pits. Local GI immune responses are regulated by specialized DCs. (vii) Secretory products of enteroendocrine cells modulate epithelial cell signaling. (viii) Likewise, components of the enteric nervous system modulate cellular and immune responses.

Hence, given the current mechanistic revelations (19), it is tempting to consider the possibility, if not likelihood, that interactions between bile acids and microbes within different compartments of the GI tract modulate inflammatory responses and the development and progression of neoplasia. Following deconjugation by bacterial hydrolases, bile acids undergo dehydroxylation and other modifications by distinct populations of gut bacteria, thereby transforming primary into secondary bile acids. Conversely, bile acids function in the gut to help shape the composition of the microbiome. As such, the potential for feedback regulation of pathways linking microbes and bile acids is extensive. Factors modulating bacterial species variation in the gut are likely to alter the ratios of different bile acid–modifying reactions and, thereby, the spectrum of different bile acids along the GI tract. There are many potential interactions between bile acids and gut microbes in this complex microenvironment. Utilizing a variety of receptor and transport mechanisms, luminal microbes and bile acids interact with healthy, inflamed, and neoplastic GI epithelial cells to modulate cell function (Figure 1).

## Unanswered questions

It might aid investigators in the field to highlight key unanswered questions raised by these considerations and the insightful work of Noto et al. (19). Although beyond the scope of the extensive work accomplished by Noto et al., we believe that several questions, based on translational potential, identify worthy areas for future exploration: (a) Does gastric neoplasia in the setting of *H*. *pylori* infection and iron deficiency result from chronic inflammation that is potentiated by the presence of selected bile acids, or do the latter have specific oncogenic effects? (b) Do similar synergistic interactions between the carcinogenic effects of bile acids and gut microbe–associated inflammation exist in other compartments of the GI tract? (c) Could bile acid sequestration or other maneuvers to alter bile acid concentration or composition attenuate colitis-associated cancer and similar cancers thought to derive from chronic GI tract inflammation?

## Conclusions and translational implications

Overall, this important study by Noto et al. (19) contributes key mechanistic insights into complex interactions between *H*. *pylori* infection, iron depletion, and bile acids in the development of gastric inflammation and neoplasia. Nonetheless, the immediate translational potential of these observations may be limited, as *H*. *pylori* infection, once diagnosed, is usually readily eliminated with antibiotics. Moreover, bile acid sequestrants are constipating and generally unpalatable, and administration must be carefully timed to avoid their adsorbing and thereby reducing the bioavailability of other medications. Thus, we believe the broader implications of this work, to understand the interaction of bile acids with different, less easily eradicated procarcinogenic microbes in other GI organs, may be even more important. We are confident that pinpointing the specific sites where bile acids interact with GI mucosal receptors will ultimately lead to greater precision in targeting these interactions. Innovative approaches, like developing structurally modified bile acid analogs, can provide additional therapies. Whereas modifications of chenodeoxycholic acid were designed to create more potent FXR agonists, like obeticholic acid (23), bile acids can also be modified to alter their interactions with GPCRs like TGR5 (24) and muscarinic receptors (25). Hence, the prospect of targeting the interaction of bile acids with GPCRs to attenuate GI inflammation and neoplasia is not only feasible, but exciting.

## Version 1 05/16/2022

Electronic publication

## References

[1] Keely SJ (2022). Contributions of bile acids to gastrointestinal physiology as receptor agonists and modifiers of ion channels. Am J Physiol Gastrointest Liver Physiol.

[2] Sheikh Abdul Kadir SH (2010). Bile acid-induced arrhythmia is mediated by muscarinic M2 receptors in neonatal rat cardiomyocytes. PLoS One.

[3] Bernstein H (2009). Bile acids as endogenous etiologic agents in gastrointestinal cancer. World J Gastroenterol.

[4] Raufman JP (2003). Activation of muscarinic receptor signaling by bile acids: physiological and medical implications. Dig Dis Sci.

[5] Yang F (2020). Structural basis of GPBAR activation and bile acid recognition. Nature.

[6] Liu R (2018). Conjugated bile acids promote invasive growth of esophageal adenocarcinoma cells and cancer stem cell expansion via sphingosine 1-phosphate receptor 2-mediated yes-associated protein activation. Am J Pathol.

[7] Shah N (2009). Muscarinic receptors and ligands in cancer. Am J Physiol Cell Physiol.

[8] Zhao CM (2014). Denervation suppresses gastric tumorigenesis. Sci Transl Med.

[9] Wang L (2018). Muscarinic acetylcholine receptor 3 mediates vagus nerve-induced gastric cancer. Oncogenesis.

[10] Schledwitz A (2021). Exploiting unique features of the gut-brain interface to combat gastrointestinal cancer. J Clin Invest.

[11] Matsuhisa T (2013). Relation between bile acid reflux into the stomach and the risk of atrophic gastritis and intestinal metaplasia: a multicenter study of 2283 cases. Dig Endosc.

[12] Tatsugami M (2012). Bile acid promotes intestinal metaplasia and gastric carcinogenesis. Cancer Epidemiol Biomarkers Prev.

[13] Li D (2020). The relationship between gastric cancer, its precancerous lesions and bile reflux: A retrospective study. J Dig Dis.

[14] Bhat AA (2018). Exposure of Barrett’s and esophageal adenocarcinoma cells to bile acids activates EGFR-STAT3 signaling axis via induction of APE1. Oncogene.

[15] Liu R (2014). Conjugated bile acids promote cholangiocarcinoma cell invasive growth through activation of sphingosine 1-phosphate receptor 2. Hepatology.

[16] Gal E (2020). Bile accelerates carcinogenic processes in pancreatic ductal adenocarcinoma cells through the overexpression of MUC4. Sci Rep.

[17] Ocvirk S, O’Keefe SJ (2017). Influence of bile acids on colorectal cancer risk: potential mechanisms mediated by diet - gut microbiota interactions. Curr Nutr Rep.

[18] Noto JM (2013). Iron deficiency accelerates Helicobacter pylori-induced carcinogenesis in rodents and humans. J Clin Invest.

[19] Noto JM (2022). Iron deficiency linked to altered bile acid metabolism promotes *Helicobacter pylori*–induced inflammation–driven gastric carcinogenesis. J Clin Invest.

[20] Raufman JP (2015). Slc10a2-null mice uncover colon cancer-promoting actions of endogenous fecal bile acids. Carcinogenesis.

[21] Cheng K (2018). Diminished gallbladder filling, increased fecal bile acids, and promotion of colon epithelial cell proliferation and neoplasia in fibroblast growth factor 15-deficient mice. Oncotarget.

[22] Sears CL (2014). Bacteroides fragilis subverts mucosal biology: from symbiont to colon carcinogenesis. J Clin Invest.

[23] Pellicciari R (2004). Bile acid derivatives as ligands of the farnesoid X receptor. Synthesis, evaluation, and structure-activity relationship of a series of body and side chain modified analogues of chenodeoxycholic acid. J Med Chem.

[24] Pellicciari R (2007). Nongenomic actions of bile acids. Synthesis and preliminary characterization of 23- and 6,23-alkyl-substituted bile acid derivatives as selective modulators for the G-protein coupled receptor TGR5. J Med Chem.

[25] Cheng K (2002). Lithocholylcholine, a bile acid/acetylcholine hybrid, is a muscarinic receptor antagonist. J Pharmacol Exp Ther.

